# Dental dam utilization by dentists in an intramural faculty practice

**DOI:** 10.1002/cre2.191

**Published:** 2019-06-10

**Authors:** Terence A. Imbery, Caroline K. Carrico

**Affiliations:** ^1^ Department of General Practice Virginia Commonwealth University School of Dentistry Richmond Virginia; ^2^ Department of Oral Health Promotion and Community Outreach, Oral Health Services Research Core, VCU Philips Institute for Oral Health Research Virginia Commonwealth University School of Dentistry Richmond Virginia; ^3^ Department of Biostatistics VCU School of Medicine Richmond Virginia

**Keywords:** dental dam, dental faculty, Isovac, operative dentistry, rubber dam

## Abstract

**Objectives:**

From casual observation of our colleagues, only a few individuals use the dental dam for operative procedures in their faculty practice. The purpose of this study was to obtain faculty perceptions of the dental dam, quantify its utilization in their intramural faculty practice, and determine the factors that influence dental dam usage.

**Material and Methods:**

A survey containing 11 questions was sent to 19 faculty members who teach full time and maintain an intramural dental practice involving operative dentistry. Thirty electronic dental health records of the 19 providers were reviewed to gather the following information from restorative procedures they completed: isolation methods, tooth location and involved surfaces, and dental restorative material.

**Results:**

Overall, dental dam was utilized for 30% of all restorative procedures and was used less than 20% of the time for placement of class II and class III composite resins. Dental dam utilization rate by general dentists was 37% and 17.6% for prosthodontists. Those general dentists with prior history of military dental practice had a utilization rate of 78.6% and nonmilitary dentists only 7.6%. Eight faculty members responded to the questionnaire for a 42% return rate. Those who practiced dentistry in the military strongly agreed that the dental dam is the standard of care, improves their quality of restorative work, and should be documented in the dental record.

**Conclusions:**

There were significantly different dental dam utilization rates between general dentists and prosthodontists and between dentists with prior military experience and those without.

## 1 INTRODUCTION

The dental dam has been the primary accepted method of isolating the operative field since its introduction by Barnum in 1864 (Reid, Callis, & Patterson, 1990). Having been recognized for providing many benefits, the dental dam's most obvious advantage is a dry and improved field of vision (Summitt, 2013). Dental dams retract and protect soft tissue from iatrogenic mishaps caused by rotary and hand instruments as well as medicaments (Chan, Myers, & Sharawy, 2007). Used properly, the dental dam protects the oral pharynx from aspiration and ingestion of unintended debris and dental instruments (Chan et al., 2007; Heling, Sommer, & Kot, 1988; Hill & Rubel, 2008a). Furthermore, it aids in the prevention and spread of infection (Cochran, Miller, & Sheldrake, 1989; Evans, Samaranayake, & Reid, 1989). Prominent dentists claim that the dental dam improves their quality and quantity of restorative procedures (Christensen, 1994; De Campos et al., 2015; Small, 1999; Small, 2002; Terry, 2005). Other advantages include facilitation of four‐handed dentistry and curtailing unnecessary patient conversation. In addition, the dental dam reduces plasma and urine mercury levels during removal of amalgam restorations (Berglund & Molin, 1991). Every state and regional licensing board in the United States require the use of the dental dam during clinical licensure examinations. In the undergraduate clinics at our school, the dental dam is the first option to isolate the operative field and is considered the standard of care. If it cannot be placed, then the alternative isolation technique is placement of the Isovac (Isolite, Santa Barbara, CA). Consisting of a bite block, tongue shield, and vacuum channel, the Isovac simultaneously provides access to two quadrants of the intraoral cavity. Purported advantages include easier placement than the dental dam, improved patient comfort, and efficiency comparable with the dental dam.

About a third of full‐time faculty who teach general dentistry and prosthodontics in the undergraduate clinics also maintain an intramural faculty dental practice. From casual observation of our colleagues, only a few faculty members use the dental dam for operative procedures. It appears that what is taught in undergraduate clinics is not practiced by faculty. Surveys ascertaining dental dam utilization by private practitioners ranged from 12% to 20% and varied by dentist, restoration type, and patient variables (Gilbert, Litaker, Pihlstrom, Amundson, & Gordan, 2010; Going & Sowinski, 1967; Hill & Rubel, 2008b; Joynt, Davis, & Schreier, 1989; Marshall & Page, 1990; Whitworth, Seccombe, Shoker, & Steele, 2000). Ireland summed it up best when he stated, “no other technique, treatment, or instrument used in dentistry is so universally accepted and advocated by recognized authorities and so ignored by practicing dentists” (Ireland, 1967). Using a dental dam was the standard of care for those who practiced dentistry in the military dental corps. Its use and reason for not using it were required documentation in dental records. Therefore, faculty members who have practiced dentistry in the military may be more proficient in dental dam application and more apt to use it in their faculty practice. Aims of this study were to obtain faculty members' opinion of the dental dam, its utilization rate in their intramural faculty dental practice, and past experience including education that would influence dental dam usage. The hypothesis is that utilization rate and opinion will vary by dental specialty and military experience.

## 2 MATERIALS AND METHODS

The University's Institutional Review Board granted exempt status for this study (IRB# HM20010348). An online survey containing 11 questions was sent to 19 full‐time dental faculty members who maintain an intramural dental faculty practice (see Appendix A). After 3 weeks, an email reminder was sent to faculty members who did not complete the questionnaire. Data from the questionnaire were collected and managed using Research Electronic Data Capture (REDCap) tools hosted by our university. Thirty electronic dental records documenting restorative procedures performed by each of the 19 dentists were reviewed. This was accomplished regardless if the provider completed the questionnaire or not. From entries in the dental record, the following information was obtained: type of restorative material used, tooth location and involved surfaces, and isolation technique (dental dam, Isovac, cotton rolls, or not recorded). We excluded the following types of restorations: class V, class IV, sealants, and preventive resin restorations. Multiple restorations placed at the same appointment were included only if they were placed in different quadrants. If the restoration was on the terminal tooth in the arch and involved multiple surfaces, it was excluded. However, if a dental dam was placed in these situations, it was included. Placement of prefabricated posts with a core, and custom cast post and cores were included. If records did not document methods used to isolate the operative field, it was assumed that a dental dam was not used, and at most, cotton roll isolation was utilized. Descriptive tables were used to illustrate the results. When appropriate, differences between faculty demographics and dental dam utilization were compared using chi‐squared tests based on raw numbers (*p* < .05). SAA Enterprise Guide v 6.1 (SAS Institute, Cary, NC) was used for statistical analysis.

## 3 RESULTS

Of the 570 records that met the inclusion criteria, 46% (*n* = 262) documented the method used for operative field isolation and 54% (*n* = 308) lacked documentation. The dental dam was used for 30% (171/570) of the procedures, the Isovac for 11% (62/570), and cotton roll isolation for 5% (29/570). For the remaining 308 (54%) procedures without any documentation regarding operative field isolation, it was assumed that at least cotton roll isolation with high‐speed aspiration may have been utilized. Therefore, cotton roll isolation most probably was used for 59% (337/570) of the procedures (see Table 1). Four of the seven prosthodontists never used a dental dam or documented the method of isolating the dental field. One prosthodontist used the dental dam for 73% (*n* = 22) of the time, another for 43% (*n* = 13), and one for 6.6% (*n* = 2) of their procedures. Two of 12 general dentists never documented their methods of isolation. One general dentist never used the dental dam but documented the use of cotton roll isolation for every procedure.

**Table 1:** The utilization rate of the three isolation techniques and those records that did not include any documentation

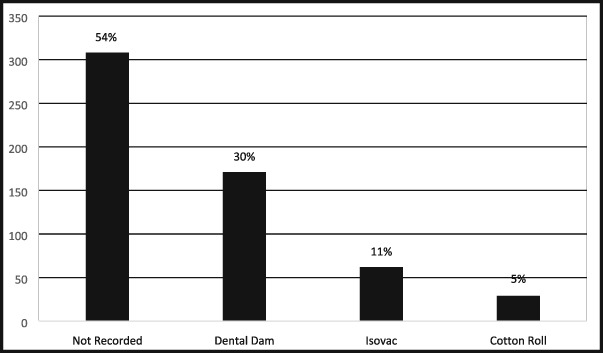

*Note*. If we assume providers who did not record isolation methods used at least cotton roll isolation, then total cotton roll usage was 59%.

General dentists had a significantly higher rate (37.2%) of dental dam utilization than prosthodontists (17.6%; *p* value <.0001; see Table 2). The rate of dental dam utilization was significantly different among general dentists who had prior military experience as compared with those who did not (78.6% vs. 7.6%; *p* value <.0001; see Table 2). The rate of dental dam utilization among dentists without military experience was only 12.6%. General dentists documented isolation methods significantly more often than prosthodontists (63.3% vs. 18.6%; *p* value <.0001).

**Table 2:** Dental dam utilization by dental specialty

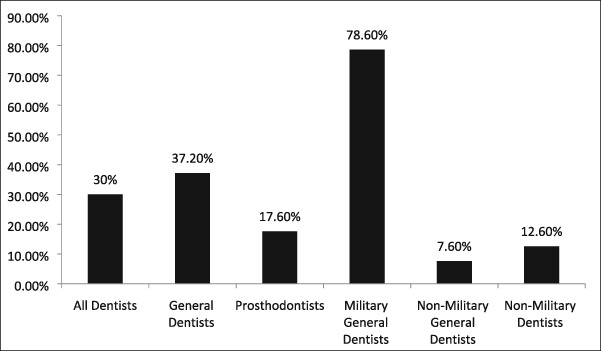

*Note*. There was a statistical difference of dental dam usage between general dentists and prosthodontists (odds ratio: 2.78, 95% confidence interval [1.83, 4.20], *p* < .0001) and between general dentists who served in the military and those general dentists who did not (odds ratio: 44.64, 95% confidence interval [23.53, 84.75], *p* < .0001).

Of the 19 dentists asked to complete the questionnaire, seven were prosthodontists and 12 were general dentists. Eight questionnaires, six from general dentists and two from prosthodontists, were completed for a return rate of 42%. The population of providers (*n* = 8) who completed the questionnaire was too small to infer any statistical analysis. Those individuals, who strongly agreed that the dental dam was the standard of care, improved the quality of their dentistry, made dentistry easier for themselves and their assistants, and believed its use required documentation in the dental record, were retired military dentists who overwhelmingly utilized the dental dam in their practice (see Likert Tables 3, 4, 5, 6, 7). Only one provider opined that the dental dam was not the standard of care and cotton roll isolation was an adequate substitute for the dental dam when placing class II composite restorations (see Likert Table 8). For some faculty, the Isovac was an acceptable alternative for the dental dam (see Likert Table 9).

**Table 3:** Dental dam is the standard of care

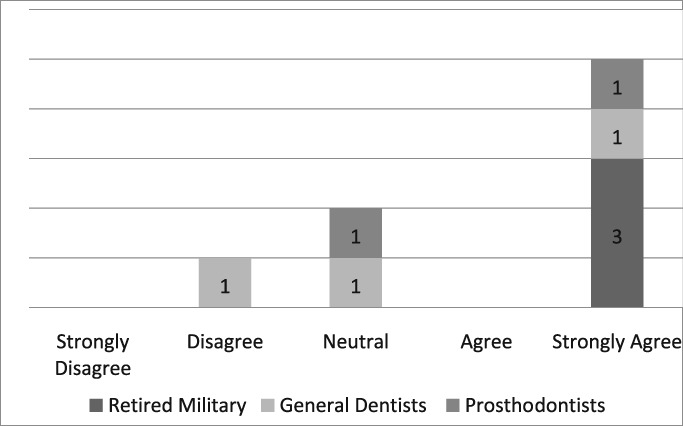

**Table 4:** Dental dam improves my quality of care

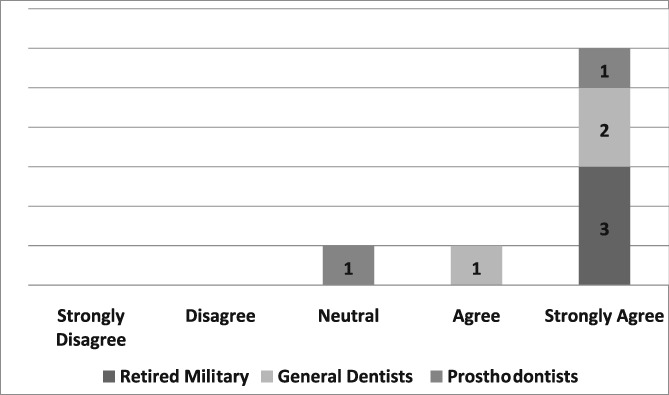

**Table 5:** Dental dam makes four‐handed dentistry easier for the dentist

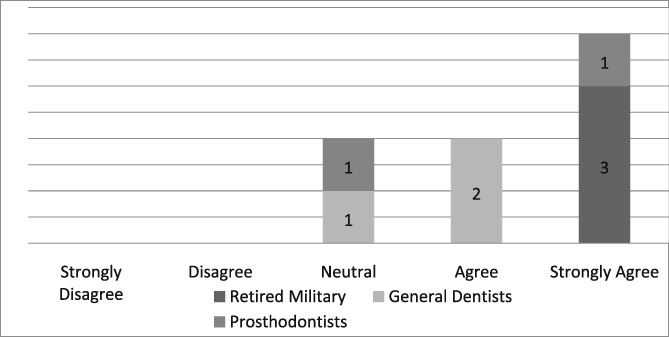

**Table 6:** Dental dam makes four‐handed dentistry easier for the dental assistant

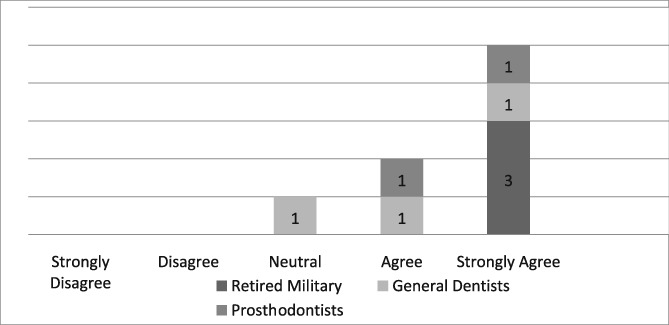

**Table 7:** Use of the dental dam should be documented

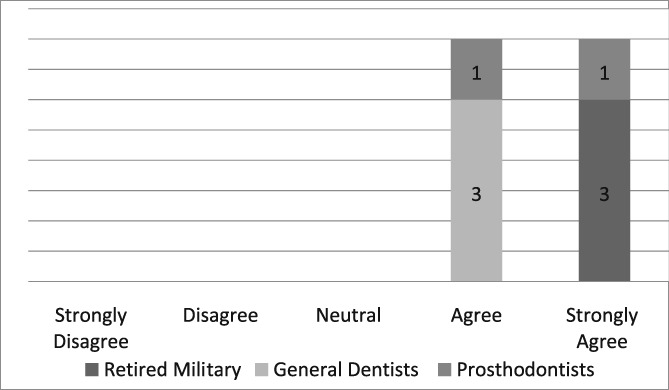

**Table 8:** Cotton roll isolation can substitute for dental dams when placing class II composites

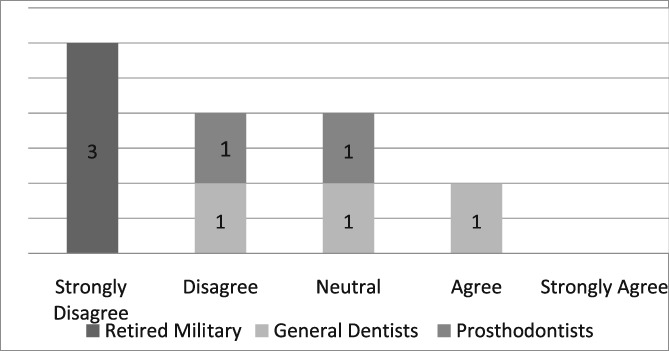

**Table 9:** Isovac can substitute for dental dams when placing class II composites

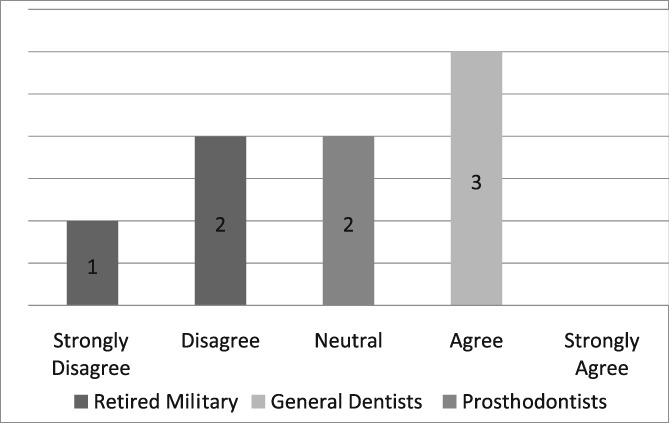

## 4 DISCUSSION

The use of the dental dam by faculty in their practice was disappointingly low but greater than practicing dentists in the general population. Surveys of dentists in nonacademic practice reported dental dam usage between 12% and 20% for restorative procedures (Gilbert et al., 2010; Going & Sowinski, 1967; Hill & Rubel, 2008b; Joynt et al., 1989; Marshall & Page, 1990; Whitworth et al., 2000). Less than half of the 570 records reviewed contained documentation describing isolation methods. One general dentist when unable to use the dental dam on four occasions explained why and documented the other methods used. Five other dentists, one prosthodontist and four general dentists, occasionally explained why the dental dam was not used.

Those who had practiced dentistry in the military were more likely to use the dental dam. In this study, five faculty members with formal advanced training in general dentistry and who obtained board certification (American Board of General Dentistry) used the dental dam 78.6% (*n* = 118/150) of the time. The use of the dental dam by these providers comprised 88% (118/134) of dental dam usage by 12 general dentists and 69% (118/171) of its overall usage by all providers. The remaining seven general dentists contributed only 9.32% (16/171) to overall dental dam usage by all dentists. A survey of dentists in the U.S. Air Force Dental Corps reported 52.4% of dentists used the dental dam 81–100% of the time for restorative work (Hagge, Pierson, Mayhew, Cowan, & Duke, 1984). In the Air Force, the dental dam is the standard of care, and its use and nonuse were required documentation in the dental health records. Eight of 19 providers, four prosthodontists and four general dentists, never used the dental dam for any procedure. However, one prosthodontist had a utilization rate of 73% and another 43%. The Isovac was overwhelmingly used by two general dentists, one who obtained a master's degree in operative dentistry and the other who completed a 1‐year residency in advanced education in general dentistry.

There were 196 class II composite resins placed with only 18.8% restored utilizing a dental dam, 17.4 % placed using an Isovac, and 63.8% using cotton isolation (see Table 10). Only 15 of 77 class III restorations were placed using a dental dam for a utilization rate of 19.5% (see Table 10). There are no other procedures that are more dependent upon optimal isolation than placement of class II and class III composite resin restorations. Contamination of the preparation with blood, saliva, and other debris may have adverse effects on the resin bond strength to dentin and enamel (Summitt, 2013). The possible resultant deleterious effects may lead to microleakage, postoperative sensitivity, recurrent caries, and loss of the restoration. The isolation methods for amalgam restorations are illustrated in Table 11. The dental dam was rarely used for class I amalgam restorations. However, for class II restorations, the dental dam was used for 46% of the restorations, Isovac for 4%, and cotton roll isolation for 56%. The dental dam and cotton roll rates were nearly identical due the extensive use of the dental dam by providers with prior military experience.

**Table 10:** I do not use the dental dam because

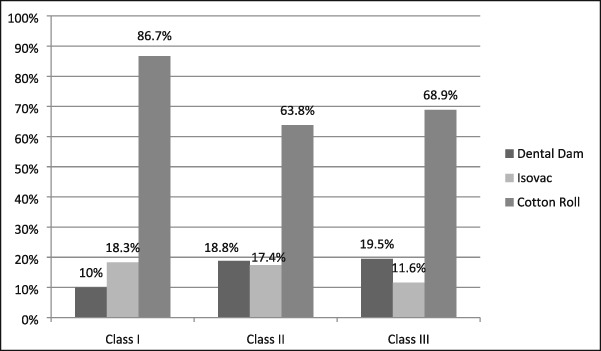

**Table 11:** The isolation methods used for class I, II, and III resin composites

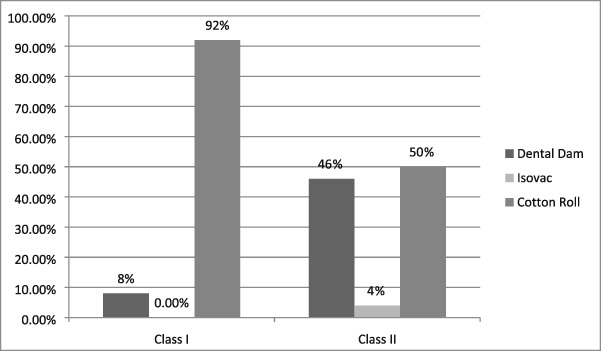

There were nine pulp exposures and nine indirect pulp capping procedures. Only in three of the nine pulp exposures was a dental dam placed. A study by de Lourdes Rodrigues Accorinte et al. reported more severe inflammatory response in teeth pulp capped without a dental dam (de Lourdes Rodrigues Accorinte, Reis, Dourado Loguercio, Cavalcanti de Araujo, & Muench, 2006). Only in two of the nine indirect pulp capping procedures was a dental dam used, one utilized the Isovac, and others utilized only cotton roll isolation. Ideally, if direct or indirect pulp capping procedures are anticipated, dental dams should be placed to prevent additional bacterial infiltration in to the root canal system (Hilton & Summitt, 2013). There were 18 custom or prefabricated post and cores placed. Only in two cases were dental dams utilized; the other 16 lacked documentation. A study by Goldfein et al. concluded that dental dam utilization during post preparation and placement significantly influenced the rate of endodontic success. Reported endodontic success rate using a dental dam during post placement was 93.4% compared with 73.6% when a dental dam was not used (Goldfein, Speirs, Finkelman, & Amato, 2013). The American Association of Endodontists position statement is as follows “Tooth isolation using the dental dam is the standard of care; it is integral and essential for any nonsurgical endodontic treatment. Only dental dam isolation minimizes the risk of contamination of the root canal system by indigenous oral bacteria” (American Association of Endodontists, n.d.). The success of endodontic treatment is dependent not only on successful debridement and obturation of the root canal system but also upon appropriate restorations that restore structural integrity of teeth. If the standard is to use the dental dam during nonsurgical root canal therapy, then it surmises that it also should be the standard when restoring endodontically treated teeth.

For those not using the dental dam but completing the questionnaire, the most frequent reasons for nonuse of the dam were their beliefs that dental assistants and alternative methods provided adequate isolation and the dental dam was disliked by patients (see Likert Table 12). Studies have documented patient acceptance of the dental dam especially if its benefits are explained (Gergely, 1989; Marshall, 2017; Stewardson & McHugh, 2002). Thus, patients may not be averse to the dental dam and may often express preference at future procedures. An operator's positive attitude and proficiency have been shown to play a role in increasing patient acceptance. Thus, the best way to increase patient acceptance is for dentists to become competent by using it more frequently. Another reason given for not using the dental dam is the time it takes to place. This slight loss of time is more than compensated by the improved working environment that reduces the frequent need for rinsing, retraction, and aspiration by dental assistants. Possibly, the most time‐consuming aspect is the time to convince oneself to use the dental dam. Therefore, many of these reasons for not using the dental dam are based on unfounded myths rather than evidenced‐based reasoning.

**Table 12:** The isolation methods used for class I and class II amalgam restorations

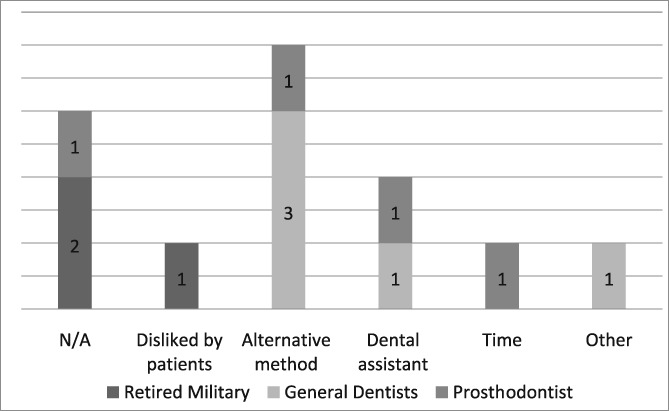

*Note*. The Isovac was not used for any class I amalgam restorations.

Several dentists who completed the survey believed that using the dental dam improves the quality of their restorative work (see Likert Table 4). Presently, there are no any long‐term clinical studies comparing the clinical performance of direct restorations placed using a dental dam compared with the Isovac. Most of the clinical studies using the Isolite and Isovac are limited to pediatric dentistry literature involving placement of sealants (Alhareky, Mermelstein, Finkelman, Alhumaid, & Loo, 2014; Collette, Wilson, & Sullivan, 2010; Feierabend, Matt, & Klaiber, 2011; Lyman, Viswanathan, & McWhorter, 2013). Raskin et al. evaluated class I and class II posterior composite resin restorations over a 10‐year period and failed to find any difference in the survival rates of restorations placed using dental dams or cotton roll isolation with high‐speed aspiration (Raskin, Sectos, Vreven, & Wilson, 2000). An extensive systematic review by the Cochrane Database of 1,213 articles concluded that there is only slight evidence to suggest that using a dental dam compared with cotton rolls improved longevity of direct restorations (Wang et al., 2016).

Other methods of isolating the operative field may be appropriate (Dahlke et al., 2012; Summitt, 2013). Poorly placed dental dams that leak are not any better than soggy cotton rolls. The Isovac along with high‐speed evacuation by a conscientious dental assistant may be equivalent to a well‐placed dental dam. Despite lack of sound confirmation that using a dental dam results in enhanced longevity of restorations, it will continue to be important medicolegally in preventing aspiration and ingestion of foreign bodies. Presently, some in the profession may doubt its benefits, but the dental dam has not lost its medicolegal importance.

Hill and Rubel proposed the following “do dental educators need to improve their approach to teaching rubber dam usage” (Hill & Rubel, 2008b). We ask, why are not dental educators using the dental dam in their own practice? Five providers strongly agreed that the dental dam is the standard of care, two were neutral and one disagreed (see Likert Table 3). If the dental dam is not the standard of care, then what is? The standard of care continuously evolves when new materials and equipment such as the Isovac are introduced (Graskemper, 2004). Is the standard of care in an undergraduate dental clinic where the dental dam is almost exclusively required different from private or faculty practice where the dental is rarely used? Is the standard of care the norm in a general dental community that rarely uses the dental dam? Unfortunately, these questions are not easily answered. If a mishap occurs when a dental dam is not used, will negligence be justified by others who practice the same kind of neglect?

A limitation of this study is that dental dams or Isovacs may have been used but were not documented in the record. We applied the following legal adage: If something is not documented in a dental health record, then it did not occur. Strengths of the study were that we were very lenient in considering which restorative procedures to include by eliminating simple and very complex procedures and not including multiple restorations placed with a single dental dam application. Therefore, clinical conditions were chosen that allowed and encouraged placement of dental dams. Due to the small population (*n* = 19) of dentists who participate in the intramural faculty, we reviewed 30 records of each provider to obtain a fair sense of dental dam utilization. The response rate on the questionnaire was lower than expected. It appeared that those dentists who used the dental dam were very supportive of letting their opinions be known. However, those who did not complete the questionnaire may be those who believe the dental dam is the standard but do not utilize it in their practice. This small population did not allow for statistical analysis of the questionnaire responses but did provide a glimpse into opinions of some. The study may have been improved if we collaborated with other institutions to determine dental dam utilizations in their intramural faculty practices.

## 5 CONCLUSION

For medicolegal purposes, providers should consider documenting the method of operative field isolation as 54% of records lacked this information. The dental dam was used for 30% of restorative procedures. General dentists were more likely to utilize the dental dam than prosthodontists. Those who tended to use the dental dam were exclusively general dentists with prior history of military service, completed a 2‐year advanced training in general dentistry, and obtain board certification.
